# Prescription Factors Associated with Medication Non-adherence in Japan Assessed from Leftover Drugs in the SETSUYAKU-BAG Campaign: Focus on Oral Antidiabetic Drugs

**DOI:** 10.3389/fphar.2016.00212

**Published:** 2016-07-20

**Authors:** Kaori Koyanagi, Toshio Kubota, Daisuke Kobayashi, Taro Kihara, Takeo Yoshida, Takamasa Miisho, Tomoko Miura, Yoshiko Sakamoto, Junichi Takaki, Takashi Seo, Takao Shimazoe

**Affiliations:** ^1^Department of Clinical Pharmacy and Pharmaceutical Care, Graduate School of Pharmaceutical Sciences, Kyushu UniversityFukuoka, Japan; ^2^Fukuoka City Pharmaceutical AssociationFukuoka, Japan

**Keywords:** leftover drugs, medication adherence, community pharmacy, pharmaceutical care, diabetes

## Abstract

**Background:** Medication adherence has an important influence on health outcomes in patients with chronic diseases. However, few studies have been performed in Japan to determine factors related to medication non-adherence.

**Objective:** The aim of this study was to identify prescription factors related to medication non-adherence by investigating patient characteristics, all prescriptions, and prescriptions for oral antidiabetic drugs (OADs).

**Methods:** A retrospective cross-sectional survey of prescription data about implementation of dosing regimen was performed at community pharmacies engaged in appropriate use of leftover drugs. We evaluated the amount of drugs originally prescribed and the reduced amount after use of leftover drugs, and then calculated prescription reduction ratio (PRR). We analyzed prescription factors contributing to non-adherence based on the PRR.

**Results:** Prescription information for 1207 patients was reviewed, revealing that patients were non-adherent to 58% of prescriptions. Lack of a drug copayment, fewer concurrent drugs, and drugs not in single-dose packaging were associated with non-adherence. Among the 1207 patients, 234 prescriptions for diabetes and 452 OAD formulations were included. Forty-seven percent of prescriptions and 29% of the formulations were non-adherent. A higher dosing frequency and preprandial administration were associated with non-adherence. Among the OADs, adherence was lower for α-glucosidase inhibitors and biguanides than for sulfonylureas.

**Conclusions:** Several factors related to patient characteristics, general drug prescriptions, and OAD prescriptions were associated with non-adherence. Further consideration will be needed to improve adherence to medication in Japan. Health care providers should perform more careful monitoring of adherence in patients with the factors identified by this study.

## Introduction

Maintaining sufficient adherence with medication is a critical issue in determining the health outcomes of patients with chronic diseases. In Japan, all citizens are covered by public health insurance and have relatively low copayments (http://www.mhlw.go.jp/bunya/iryouhoken/iryouhoken01/dl/01_eng.pdf). Most patients with chronic diseases consult their family doctor regularly, and drugs are prescribed depending on the consultation schedule. Therefore, if medication adherence is sufficiently high, patients with chronic diseases should not have leftover drugs. However, medication non-adherence results in home storage of leftover drugs for various reasons (Kardas et al., 2013), both in Japan (Kutsuma et al., 2004; Hatanaka et al., 2009) and in other countries (Ruhoy and Daughton, 2008; Kevin, 2010).

This situation suggests a possibility of reducing medical expenditure. For instance, a previous study indicated that appropriate reuse of leftover drugs could contribute to reducing medical costs (Kutsuma et al., 2004). Furthermore, non-adherence is linked to increased hospitalization (Juarez et al., 2013; Shin et al., 2013), higher all-cause mortality (Chowdhury et al., 2013; Shin et al., 2013), and higher healthcare costs (Egede et al., 2012; Wong et al., 2014). Because of these findings, community pharmacists have been obliged to check and count patients' leftover drugs from April 2012 in Japan.

The Fukuoka City Pharmaceutical Association started the SETSUYAKU-BAG campaign in collaboration with Kyushu University from June 2012. This campaign has three aims: (1) to reduce medical expenditure by promoting appropriate reuse of leftover drugs; (2) to ascertain and improve adherence with medication by educating patients about leftover drugs; (3) to avoid the risk of incorrect use of accumulated leftover drugs by patients. Our previous research (Koyanagi et al., 2013) assessed the current situation of leftover drug retention by adult outpatients, and proved that community pharmacists could reduce medical costs by appropriate reuse of such drugs. Based on our findings, we estimated the possible reduction of medical costs for all Japan in 1 year. However, we only surveyed leftover drugs, so we were not able to clarify the reduction rate for original prescriptions, and the prescription factors for medication non-adherence.

Understanding the factors contributing to non-adherence has the potential to guide efforts and interventions for improving the adherence of patients to their medications. In Japan, several studies about medication adherence and non-adherence have already been performed (Kitagawa et al., 2012; Matsumura et al., 2012). However, these studies were relatively small in scale and were limited to particular diseases or drugs. Many studies about medication adherence using pharmacy claim database have been performed in other countries (Chapman et al., 2005; Han et al., 2012; Chowdhury et al., 2013; Sattler et al., 2013; Kirkman et al., 2015). The medication possession ratio (MPR) or the proportion of days covered (PDC) are common methods of measuring medication adherence based on pharmacy refills or claims data for chronic conditions (Andrade et al., 2006). In Japan, we do not have a refill prescription system, and it is difficult to utilize the pharmacy claims database. Therefore, a different method is necessary to assess medication adherence in Japan.

In the present study, we collected both original prescriptions and prescription adjustment information of patients with chronic disease who regularly consult their family doctor from 127 pharmacies for a year through the SETSUYAKU-BAG campaign. Adherence to medications is defined as the process by which patients take their medication as prescribed, further divided into three quantifiable phase: initiation, implementation, and discontinuation (Vrijens et al., 2012). We assessed prescription factors related to non-adherence about implementation of dosing regimen for all oral drugs and also analyzed factors related to non-adherence for oral antidiabetic drugs (OADs). We evaluated the extent of non-adherence by using a different method to assess medication adherence, which was based on prescription reduction ratio (PRR); the reduction ratio of the amount of drugs in the original prescriptions by appropriate utilization of leftover drugs.

## Materials and methods

### Study design

**Figure 1:**
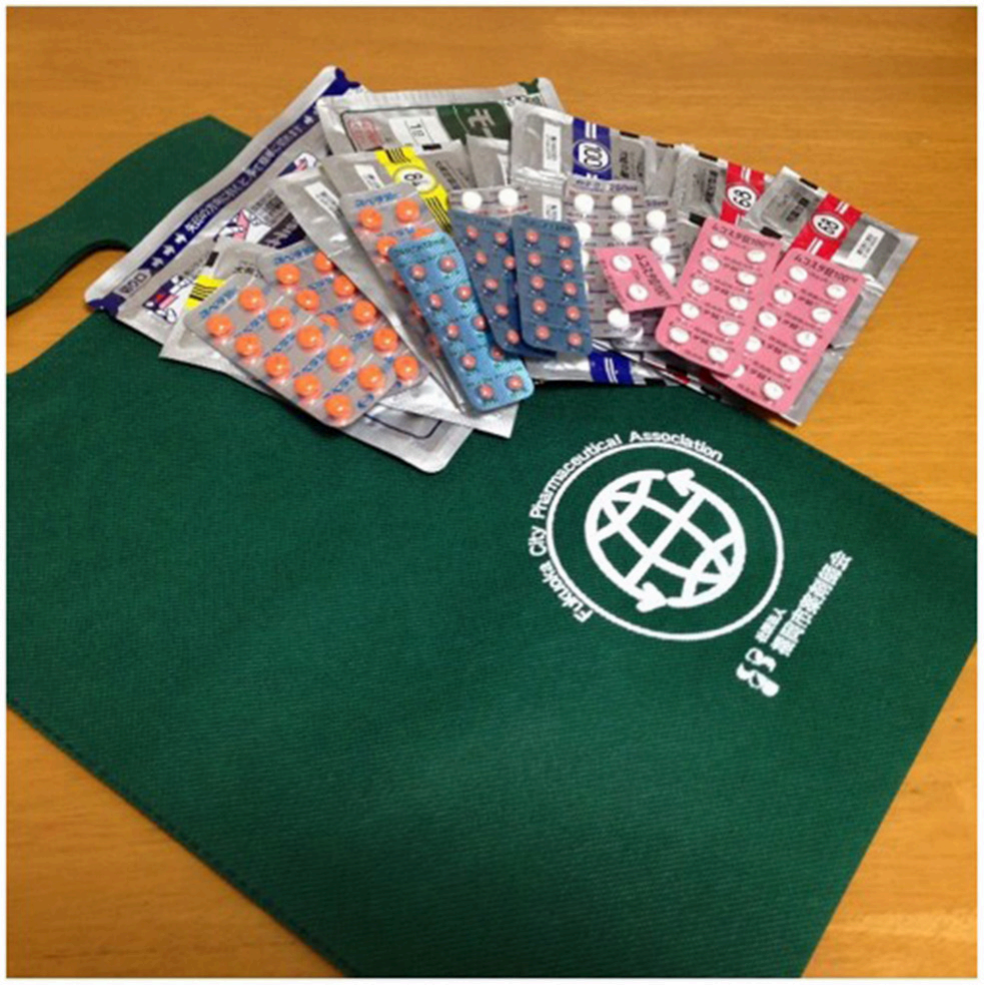
**A SETSUYAKU-BAG and leftover drugs brought to pharmacies by patients**.

This study was performed in Fukuoka city (1.5 million inhabitants) in southern Japan. Between February 1, 2013 and January 31, 2014, we implemented this study in collaboration with Kyushu University at 645 community pharmacies that were the Fukuoka City Pharmaceutical Association members. Pharmacists explained the aims and procedures of this study to eligible patients and obtained informed consent, after which they documented the details of the explanation provided and acquisition of consent in the medication records. Pharmacists also gave each patient a bag (SETSUYAKU-BAG), and asked the patient to put all leftover drugs in this bag and bring them the next time they came to fill a prescription. An SETSUYAKU-BAG and leftover drugs brought to pharmacies by patients is shown in Figure 1. When a patient brought leftover drugs to the community pharmacy, the pharmacist inspected the medications. If they could still be used, the pharmacist advised the patient to utilize the leftover drugs before the expiration date. After acquiring the patient's consent, the pharmacist requested the patient's doctor to adjust the prescription(s) accordingly. The pharmacist wrote the patient's age, sex, and copayment for medication on a SETSUYAKU-BAG campaign information sheet (Supplementary Figure 1) and also wrote details of adjustments to medication amounts on a copy of each prescription (Supplementary Figure 2). After deleting personal information (name, birth date, insurance number, etc.), the pharmacist sent the information sheet and prescription copies to Kyushu University. This study was approved by the Ethics Committees of both the Fukuoka City Pharmaceutical Association and Kyushu University.

### Data analysis

To assess the non-adherence for oral drugs, we excluded, (1) topical medications, (2) drugs taken as needed, (3) prescriptions outside the national health insurance scheme. We totaled the amount of drugs according to the original prescriptions and the reduced amount after adjustment for leftover drugs. Among patients with diabetes, we selected those with prescriptions for OADs, which were defined according to standard commodity classification of Japan (http://www.soumu.go.jp/main_content/000294493.pdf). Drug amounts were counted by using a drug unit (tablet, capsule, etc.). We evaluated the extent of non-adherence by calculating the PRR after use of leftover drugs as follows:

PRR (%) = amount of drugs reduced after adjustment for leftover drugs/amount of drugs originally prescribed.

A MPR of 0.8 or higher is commonly used as a threshold to define adherence (Hess et al., 2006; Karve et al., 2009). Therefore, we considered patients to be non-adherent if the PRR was >0.2, implying that >20% of their prescribed drugs had not been used.

A logistic regression analysis was performed to evaluate the association of medication non-adherence, and odds ratio (OR), 95% confidence interval (CI) and C-statistic were calculated. We separately analyzed factors for all oral drugs and for OADs. For all oral drugs, we adjusted the total number of concurrent drugs, patient copayment, and nonuse of single-dose packaging. For OADs, we adjusted the total number of concurrent drugs, number of concurrent OADs, OAD prescription days, and nonuse of single-dose packaging. For OAD utilization, we adjusted total number of concurrent drugs.

Data are shown as the mean ± SD (range; median). Statistical significance was assumed at *p* < 0.05, and precision of estimates was assessed from the 95% confidence interval. All statistical analyses were performed using JMP pro 11 software (SAS Institute Inc., Cary, NC).

## Results

We collected data on 1376 prescriptions from 127 pharmacies. A total of 1207 prescriptions (for 1207 patients) met the criteria for this study. The patients included 532 males and 675 females with a mean age of 68.3 ± 15.8 (range: 0.3–99) years. The characteristics of the patients and their prescriptions are shown in Table 1. The mean number of concurrent drugs and prescription days were 5.6 ± 2.9 (range: 1–17) drugs and 27.7 ± 14.1 (range: 3–98) days, respectively. The total amount of drugs originally prescribed was 298,020 doses and this was reduced by 80,902 doses after adjustment for utilization of leftover drugs, which was a 27% reduction.

**Table 1:** **Characteristics of the patients and their prescriptions**.

**Patient characteristics**	**All oral drugs**	**OADs**
Patients, n	1207	234
Age (years)	68.3 ± 15.8 (range: 0.3–99; median 70)	70.1 ± 11.3 (range: 28–89; median 70)
**SEX, N (%)**		
Male	532 (44.1)	142 (60.7)
Female	675 (55.9)	92 (39.3)
**PATIENT COPAYMENT, N (%)**		
0%	143 (11.8)	13 (5.6)
10%	491 (40.7)	99 (42.3)
30%	573 (47.5)	122 (52.1)
Total number of concurrent drugs	5.6 ± 2.9 (range: 1–17; median 5)	1.9 ± 0.9 (range: 1–5; median 2)
**CONCURRENT OADs**		
Single, n (%)	–	90 (38.5)
Multiple, n (%)	–	144 (61.5)
Prescription days	27.7 ± 14.1 (range: 3–98; median 28)	31.4 ± 12.5 (range: 7–90; median 30)
Total number of formulations, n	6744	452
Total amount of original prescription drugs, n	298,020	26,357.5
Total amount of reduction drugs, n (%)	80,902 (27.1)	5696 (22.6)
Mean amount of original prescription drugs	246.9 ± 190.7 (range: 5–2079; median 210)	112.6 ± 95.6 (range: 7–510; median 84)
Mean amount of reduction drugs	67.0 ± 87.5 (range: 1–1744; median 42)	25.5 ± 39.5 (range: 0–231; median 7.3)
Mean PRR (%)	29.3 ± 22.1 (range: 0.2–100; median 24.8)	19.4 ± 25.4 (range: 0–100; median 9.0)

*Data are represented as the mean ± SD (range; median)*.

*OADs, oral antidiabetic drugs; PRR, prescription reduction ratio*.

Factors associated with non-adherence for all oral drugs are shown in Table 2. Among the 1207 patients, 695 (58%) met the criteria for non-adherence. Seventy-eight percent of patients were 60 years old or older, 56% were female, and 48% were required to make a 30% copayment for medical costs. Seventy-five percent of prescriptions were for more than four concurrent drugs, 82% were for < 30 days, and 15% were for drugs in single-dose packaging. Multivariate logistic regression analysis demonstrated that several factors were associated with non-adherence. The risk of non-adherence was higher in patients who did not have to pay drug copayment (0%: OR = 1.67, CI = 1.12–2.50), and were prescribed fewer concurrent drugs (< 4: OR = 3.41, CI = 2.43–4.82; 4–6: OR = 1.83, CI = 1.39–2.42). Patients with single-dose packaging showed a lower risk of non-adherence (OR = 0.63, CI = 0.44–0.89) than patient without single-dose packaging. The C-statistic was 0.637–0.641.

**Table 2:** **Factors contributing to non-adherence for all oral drugs**.

	**Non-adherent (PRR > 0.2) *n* = 695 (57.6)**	**Adherent (PRR ≤ 0.2) *n* = 512 (42.4)**	**Total *n* = 1207 (100)**	**Odds ratio (95% CI)**	***P* value**	**C-statistic**
**PATIENT FACTORS**						
**Age, n (%)**						
<40	48 (6.9)	29 (5.7)	77 (6.4)	1.13 (0.64–2.00)	0.682	0.637
40–59	113 (16.3)	73 (14.3)	186 (15.4)	1.02 (0.66–1.59)	0.913	–
60–74	266 (38.3)	184 (35.9)	450 (37.3)	1.02 (0.74–1.40)	0.919	–
≥75	268 (38.6)	226 (44.1)	494 (40.9)	1.00	NA	–
**Sex, n (%)**						
Male	300 (43.2)	232 (45.3)	532 (44.1)	0.90 (0.71–1.14)	0.395	0.638
Female	395 (56.8)	280 (54.7)	675 (55.9)	1.00	NA	–
**Patient copayment, n (%)**						
0%	89 (12.8)	54 (10.5)	143 (11.8)	1.67 (1.12–2.50)	0.011^*^	0.637
10%	270 (38.8)	221 (43.2)	491 (40.7)	1.10 (0.85–1.43)	0.452	–
30%	336 (48.3)	237 (46.3)	573 (47.5)	1.00	NA	–
**PRESCRIPTION FACTORS**						
**Total number of concurrent drugs, n (%)**						
<4	224 (32.2)	82 (16.0)	306 (25.4)	3.41 (2.43–4.82)	<0.001^*^	0.641
4–6	296 (42.6)	203 (39.6)	499 (41.3)	1.83 (1.39–2.42)	<0.001^*^	–
≥7	175 (25.2)	227 (44.3)	402 (33.3)	1.00	NA	–
**Prescription days, n (%)**						
≤30	565 (81.3)	426 (83.2)	991 (82.1)	1.00	NA	0.638
>30	130 (18.7)	86 (16.8)	216 (17.9)	1.12 (0.82–1.53)	0.483	–
**Single-dose packaging, n (%)**						
Yes	75 (10.8)	106 (20.7)	181 (15.0)	0.63 (0.44–0.89)	0.008^*^	0.637
No	620 (89.2)	406 (79.3)	1026 (85.0)	1.00	NA	–

*The odds ratio (OR) and 95% confidence interval (CI) were calculated to predict the factors associated with non-adherence (PRR > 20%). Logistic regression models were adjusted for the total number of concurrent drugs, patient copayment, and nonuse of single-dose packaging*.

* *Statistically significant at the 5% level. Adherence status: number (%)*.

*PRR, prescription reduction ratio*.

Characteristics of the diabetic patients and their prescriptions are also shown in Table 1. There were 234 OAD prescriptions for 234 patients, including 142 males and 92 females with a mean age of 70.1 ± 11.3 (range: 28–89) years, and the total number of formulations was 452. The mean number of concurrent OADs and prescription days were 1.9 ± 0.9 (range: 1–5) drugs and 31.4 ± 12.5 (range: 7–90) days, respectively. The total amount of OADs before adjustment was 26,357.5 doses and the reduction was 5696 doses (23%).

Factors associated with non-adherence to treatment with OADs are shown in Table 3. Among the 234 patients, 110 (47%) met the criteria for non-adherence. Over 84% of patients were 60 years old or older, 39% were female, and 52% had a 30% copayment. There were 90 prescriptions for a single OAD and 144 for multiple OADs. Seventy-four percent were prescribed for < 30 days and 18% of prescriptions were for drugs in single-dose packaging. Based on the results of multivariate logistic regression, no factor was associated with non-adherence. The C-statistic was 0.664–0.680. Factors associated with OAD non-adherence in relation to dosage and drug class are shown in Table 4. Among 452 formulations, 132 (29%) met the criteria of non-adherence. Fifty-eight percent were for once-daily dosing, 72% were taken postprandially, and 33% of the formulations were for dipeptidyl peptidase-4 inhibitors (DPP4I). The risk of non-adherence increased in drugs prescribed with a higher dosing frequency (twice: OR = 4.82, CI = 2.81–8.36; three times: OR = 8.64, CI = 5.10–14.92). Preprandial administration showed a higher risk of non-adherence (OR = 2.68, CI = 1.73–4.16) than postprandial administration. Compared to sulfonylureas (SU), α-glucosidase inhibitors (αGI), and biguanides (BG) showed a higher risk of non-adherence (αGI: OR = 6.86, CI = 3.54–13.72; BG: OR = 3.83, CI = 2.02–7.43). The C-statistic was 0.643–0.743.

**Table 3:** **Factors contributing to non-adherence for OADs**.

	**Non-adherent (PRR > 0.2) *n* = 110 (47.0)**	**Adherent (PRR ≤ 0.2) *n* = 124 (53.0)**	**Total *n* = 234 (100)**	**Odds ratio (95% CI)**	***P* value**	**C-statistic**
**PATIENT FACTORS**						
**Age, n (%)**						
<60	22 (20.0)	15 (12.1)	37 (15.8)	1.38 (0.58–3.34)	0.471	0.680
60–74	50 (45.5)	59 (47.6)	109 (46.6)	0.99 (0.52–1.89)	0.985	–
≥75	38 (34.5)	50 (40.3)	88 (37.6)	1.00	NA	–
**Sex, n (%)**						
Male	71 (64.5)	71 (57.3)	142 (60.7)	1.00	NA	0.680
Female	39 (35.5)	53 (42.7)	92 (39.3)	0.81 (0.46–1.41)	0.447	–
**Patient copayment, n (%)**						
0%	7 (6.4)	6 (4.8)	13 (5.6)	1.13 (0.32–4.07)	0.849	0.675
10%	43 (39.1)	56 (45.2)	99 (42.3)	0.88 (0.49–1.61)	0.687	–
30%	60 (54.5)	62 (50.0)	122 (52.1)	1.00	NA	–
**PRESCRIPTION FACTORS**						
**Number of concurrent OADs, n (%)**						
1	48 (43.6)	42 (33.9)	90 (38.5)	1.00	NA	0.673
2	40 (36.4)	47 (37.9)	87 (37.2)	0.69 (0.37–1.28)	0.235	–
3–5	22 (20.0)	35 (28.2)	57 (24.4)	0.57 (0.28–1.15)	0.117	–
**Prescription days, n (%)**						
≤ 30	83 (75.5)	90 (72.6)	173 (73.9)	1.00	NA	0.664
>30	27 (24.5)	34 (27.4)	61 (26.1)	0.89 (0.48–1.65)	0.709	–
**Single-dose packaging, n (%)**						
Yes	12 (10.9)	31 (25.0)	43 (18.4)	0.58 (0.25–1.31)	0.191	0.674
No	98 (89.1)	93 (75.0)	191 (81.6)	1.00	NA	–

*The odds ratio (OR) and 95% confidence interval (CI) were calculated to predict the factors associated with non-adherence (PRR > 20%). Logistic regression models were adjusted for the total number of concurrent drugs, number of concurrent OADs, OAD prescription days, and nonuse of single-dose packaging*.

*Adherence status: number (%)*.

*OADs, oral antidiabetic drugs; PRR, prescription reduction ratio*.

**Table 4:** **Factors contributing to OAD non-adherence associated with dosage and drug class**.

	**Non-adherent (PRR > 0.2) *n* = 132 (29.2)**	**Adherent(PRR ≤ 0.2) *n* = 320 (70.8)**	**Total = 452 (100)**	**Odds ratio (95% CI)**	***P* value**	**C-statistic**
**DOSING FREQUENCY, N (%)**						
Once	35 (26.5)	225 (70.3)	260 (57.5)	1.00	NA	0.743
Twice	40 (30.3)	53 (16.6)	93 (20.6)	4.82 (2.81–8.36)	<0.001^*^	–
Three times	57 (43.2)	42 (13.1)	99 (21.9)	8.64 (5.10–14.92)	<0.001^*^	–
**ADMINISTRATION TIME, N (%)**						
Preprandial	56 (42.4)	70 (21.9)	126 (27.9)	2.68 (1.73–4.16)	<0.001^*^	0.643
Postprandial	76 (57.6)	250 (78.1)	326 (72.1)	1.00	NA	–
**TYPE OF OAD, N (%)**						
SU	23 (17.4)	100 (31.3)	123 (27.2)	1.00	NA	0.714
BG	35 (26.5)	38 (11.9)	73 (16.2)	3.83 (2.02–7.43)	<0.001^*^	–
DPP4I	29 (22.0)	119 (37.2)	148 (32.7)	1.04 (0.57–1.93)	0.889	–
αGI	40 (30.3)	25 (7.8)	65 (14.4)	6.86 (3.54–13.72)	<0.001^*^	–
Others	5 (3.8)	38 (11.9)	43 (9.5)	0.57 (0.18–1.50)	0.264	–

*The odds ratio (OR) and 95% confidence interval (CI) were calculated to predict the factors associated with non-adherence (PRR > 20%). Logistic regression models were adjusted for the total number of concurrent drugs*.

* *Statistically significant at the 5% level*.

*Adherence status: number (%)*.

*OADs, oral antidiabetic drugs; PRR, prescription reduction ratio; SU, sulfonylureas; BG, biguanides; DPP4I, dipeptidyl peptidase-4 inhibitors; αGI, α-glucosidase inhibitors*.

## Discussion

In the present study, we investigated leftover drugs and adjusted the prescriptions of patients by utilizing these drugs. We assessed prescription factors related to medication non-adherence about implementation of dosing regimen by the PRR. A high PRR meant that the patient had a larger number of leftover drugs, indicating non-adherence. Analysis of factors associated with non-adherence for all oral drugs showed that 58% of patients were non-adherent and several factors were associated with medication non-adherence. Lack of a drug copayment and fewer concurrent drugs were associated with non-adherence, while single-dose packaging enhanced adherence. Among patients using antidiabetic drugs, 47% were non-adherent, but no factors associated with medication adherence were identified. Regarding OAD utilization, 29% of the formulations met the criteria for non-adherence. Patients with a lower dosing frequency for their medications clearly showed better adherence, while preprandial administration was associated with worse adherence. In addition, adherence to treatment with αGI or BG was lower than for treatment with SU.

Based on our prescription adjustment information, the characteristics of the patients were varied and 75% of the prescriptions for oral drugs were for four or more concurrent drugs. Rolnick et al. examined medication adherence in patients with multiple diseases and stated that the adherence rate varied from 32 to 75% (Rolnick et al., 2013). Chapman et al. reported that the long-term adherence rate was only 36% for both antihypertensive therapy and lipid-lowering therapy (Chapman et al., 2005). In prior studies of diabetic patients, medication adherence rates have ranged from 45 to 78% (Curkendall et al., 2013; Guénette et al., 2013; Kirkman et al., 2015; Simard et al., 2015; Tunceli et al., 2015). Our findings seem to be consistent with the results of these studies in other countries. Japan has a different sociological and cultural background compared to Europe and the US where most of the research on medication adherence were performed. Although we were not able to assess the sociological and cultural custom of the patients, it might be suggested that non-adherence was principally dependent on the relation of the patients' therapeutic program and was not very deeply influenced by the ethical norms of the society. Further consideration will be needed to yield any findings about these points.

We found that patients who did not have to make a drug copayment were less likely to show adherence. Previous studies have suggested that high drug costs (Malmenäs et al., 2013; Kirkman et al., 2015; Simard et al., 2015; Tunceli et al., 2015) and lower incomes (Rolnick et al., 2013; Kirkman et al., 2015) were associated with non-adherence. In Japan, as one of the solutions to this problems, the government has set different patient copayment rates depending on a person's circumstances (ordinary citizen: 30%, elderly person: 10%, low income person: no copayment). The lack of co-payment seems to stimulate or oblige the patients to save up the drugs even if they do not intend to use it, and might have led to reduced appreciation of the importance of medication. Pharmacists should take account of this tendency and give more careful and frequent observation on these patients. Furthermore, it is desirable that pharmacists confirm the necessity of their medication, evaluate their dosing regimen and share the result with the doctors. We think pharmacists could contribute to optimize the patients' prescription in cooperation with doctors.

Regarding the effect of the total number of concurrent drugs on adherence, contradictory results have been reported. In our study, a higher total number of concurrent drugs was associated with better adherence and this was also found by some large-scale studies of adherence to medication (Guénette et al., 2013; Kirkman et al., 2015; Tunceli et al., 2015). However, Chapman et al. reported that patients were more likely to be adherent if they took fewer medications (Chapman et al., 2005).

We also found that single-dose packaging of medication enhanced adherence, a result that is in agreement with another report (Hatanaka et al., 2009). There have been few studies about the influences of single-dose packaging on medication adherence in Japan, but it is possible that better adherence associated with a higher total number of medications might have been influenced by single-dose packaging. In this study, the use of single-dose packaging increased along with the total number of drugs prescribed (< 4: 2.6%, 4–6: 10.0%, ≥7: 30.6%). Thus, single-dose packaging tended to increase along with the total number of medications and this form of packaging for medications might be an effective technique for enhancing adherence, especially in polypharmacy.

A lower dosing frequency was clearly associated with better adherence. Comparing the medication adherence of diabetic patients between once-daily dosing and twice-daily dosing, Tunceli et al. and Malmenäs et al. reported that twice-daily dosing resulted in 18 and 33% lower adherence than once-daily dosing, respectively. In these studies, once-daily dosing was not compared with thrice-daily dosing (Malmenäs et al., 2013; Tunceli et al., 2015). Our study revealed larger differences of adherence in relation to dosing frequency, which were 4.8-fold for once vs. twice and 8.6-fold for once vs. three-times.

Preprandial administration was associated with lower medication adherence compared to postprandial administration. Masuda et al. mentioned that one reason for non-adherence to αGI therapy is the need to take these drugs before meals (Masuda et al., 2011). While little has been reported about the influence of preprandial dosing on medication adherence, αGI are frequently prescribed in Japan and we found that αGI accounted for 14% of all the OAD formulations. As long as this situation continues in Japan, it seems reasonable for pharmacists to apply more careful adherence monitoring to these drugs.

Among the OADs, adherence was less likely with αGI and BG than with SU. An effect of OAD class on adherence has been found in several previous studies. Simard et al. stated that adherence was lower with αGI than other OADs, while adherence was higher with BG than SU, αGI, or meglitinides (Simard et al., 2015). A prospective study performed in the United Kingdom assessed 60 patients for 2 months and showed that adherence was lower with BG than SU (White et al., 2012). Curkendall et al. stated that patients taking SU or thiazolidinediones (TZD) were less likely to be adherent than those taking saxagliptin (a DPP4I) (Curkendall et al., 2013). A high dosing frequency and/or preprandial administration is required with αGI and BG. In Japan, the standard regimen for αGI is administration three times daily just before each meal, while BG are taken twice or three times daily before or after meals. Based on these regimens, our results seem understandable. We think there are three reasons for poor adherence to αGI therapy: (1) administration just before each meal; (2) dosing three times a day; and (3) gastrointestinal adverse effects. In Japan, metformin might be less commonly used than in Europe and the USA, since we found it was prescribed in 16% of all the OAD formulations. Along with the high dosing frequency of BG, we think this might be one of the reasons for non-adherence to BG therapy. According to our findings, DPP4I and other drugs (including TZD) showed no significant differences to SU with respect to non-adherence.

The present study differed from previous studies in the following points. We attempted to use the PRR which was different from standard method to assess medication non-adherence. To determine drug utilization, we inspected the actual leftover drugs rather than performing a database survey, and calculated PRR. The MPR is the ratio of the number of prescribed days to the number of days for which the drug should have been prescribed during the equivalent period. On the other hand, the PRR is the reduction ratio of the amount of drugs in the original prescriptions by appropriate utilization of leftover drugs. Thus, the PRR expresses the reverse concept to the MPR. In Japan where a refill prescription system is not adopted, fewer medication adherence studies have been performed by using the pharmacy claims database than in other countries because of the difficulty in mining this database. In fact, Andrade et al. mentioned that medication adherence study using such a database had not been performed in Japan (Andrade et al., 2006). In Japan, all community pharmacists check and count patients' leftover drugs as part of their regular duties. This means that assessment of medication adherence by calculating PRR might be useful in Japan. Furthermore, our findings seem to be consistent with the results of medication adherence which were evaluated based on standard index (Chapman et al., 2005; Curkendall et al., 2013; Guénette et al., 2013; Malmenäs et al., 2013; Rolnick et al., 2013; Kirkman et al., 2015; Simard et al., 2015; Tunceli et al., 2015). Although both actual drug consumption and the quantity prescribed during the investigation period should be surveyed as the optimum method of assessing medication adherence, until access, and utilization of the pharmacy claims database become easier, we think it seems reasonable to use PRR as a simple index for evaluating non-adherence in Japan.

Our study had several limitations. First, our sample size was smaller than in other studies (Chapman et al., 2005; Curkendall et al., 2013; Guénette et al., 2013; Malmenäs et al., 2013; Rolnick et al., 2013; Kirkman et al., 2015; Simard et al., 2015; Tunceli et al., 2015). Regarding the effects of age and sex on adherence, previous studies have provided contradictory results. Several studies have shown that older patients (Curkendall et al., 2013; Guénette et al., 2013; Malmenäs et al., 2013; Rolnick et al., 2013; Kirkman et al., 2015; Simard et al., 2015; Tunceli et al., 2015) and male patients (Chapman et al., 2005; Curkendall et al., 2013; Malmenäs et al., 2013; Rolnick et al., 2013; Kirkman et al., 2015; Tunceli et al., 2015) had higher adherence. In our study, there was no effect of these factors, but it might be possible that the sample size influenced these results. Second, we used a cross-sectional design to investigate leftover drugs and the study was also limited to one city in Japan, so we cannot exclude the possibility that different results might be obtained if the same survey was performed at another time or place with different patients. The evaluation period of non-adherence depends on the prescription interval when the PRR is used, and it was shorter than in previous studies (Chapman et al., 2005; Curkendall et al., 2013; Guénette et al., 2013; Malmenäs et al., 2013; Rolnick et al., 2013; Kirkman et al., 2015; Simard et al., 2015; Tunceli et al., 2015), which set an evaluation period of 1 year or more. Thus, it seems possible that our findings are less precise than those of the other studies. Third, the PRR may overestimate medication non-adherence. This study investigated patients who brought prescriptions and leftover drugs to pharmacies. We only collected the prescriptions of patients with leftover drugs and we did not include patients who were adherent without leftover drugs. It might be a possible biases because of only counting patients who returned leftover drug. In Japan, the ratio of patients not retaining leftover drugs was reported to be ~44% in 2013 by Ministry of Health, Labour and Welfare, Japan (http://www.mhlw.go.jp/file/05-Shingikai-12404000-Hokenkyoku-Iryouka/0000092092.pdf). On the other hand, we cannot exclude the possibility that there were more patients who did not bring their leftover drugs. Finally, although medication adherence should be evaluated about patient factors (including patients' beliefs and mood), clinician factors, and treatment factors (Stack et al., 2008, 2011; Gadkari and McHorney, 2012), we were not able to assess these factors. In this study, we were only able to collect prescription data, and we could not obtain the information about clinicians and patients' treatment because of Japanese prescription form. In addition, we did not perform questionnaire survey for patients, so it was difficult to assess the elements about patients' beliefs and mood.

In this study, we assessed prescription factors associated with medication non-adherence about implementation of dosing regimen by the PRR. Our findings fit reasonably with the results of previous studies. The PRR is not a standard index of medication adherence, but it might be able to measure actual medication taking more accurately. Further consideration of adherence to medication will be needed in Japan, but it seems reasonable for health care providers (such as pharmacists) to perform more careful adherence monitoring in the following situations: (1) no drug copayment and (2) drugs taken frequently and/or before meals. In addition, they should not assume that patients who use fewer medications are more likely to be adherent. Furthermore, when patients have a complicated regimen with multiple drugs, single-dose packaging might be one effective method of enhancing adherence.

## Author contributions

All authors contributed to the study design. All authors participated in collecting and interpreting the data. KK analyzed data and drafted the manuscript. TM and YS confirmed the analyzed data. DK analyzed data and revised the manuscript. TK and TS revised the manuscript. All authors reviewed and approved the final manuscript.

## Funding

This work was supported by JSPS KAKENHI Grant Number 16K08873.

### Conflict of interest statement

The authors declare that the research was conducted in the absence of any commercial or financial relationships that could be construed as a potential conflict of interest.
