# Supplementary material for: Prescription Factors Associated with Medication Non-adherence in Japan Assessed from Leftover Drugs in the SETSUYAKU-BAG Campaign: Focus on Oral Antidiabetic Drugs
Source: Front Pharmacol. 2016 Jul 20;7:212. doi: 10.3389/fphar.2016.00212 (PMC4951482; doi:10.3389/fphar.2016.00212)
Supplement: Supplementary file 1 [file Image1.PDF]

Supplementary Material

Factors associated with medication non-adherence in Japan assessed from leftover drugs in the SETSUYAKU-BAG campaign: Focus on oral antidiabetic drugs

Kaori Koyanagi[1, 2], Toshio Kubota[1], Daisuke Kobayashi[1*], Taro Kihara[2], Takeo Yoshida[2], Takamasa Miisho[2], Tomoko Miura[1], Yoshiko Sakamoto[1], Junichi Takaki[2], Takashi Seo[2], and Takao Shimazoe[1]

*** Correspondence:**
Daisuke Kobayashi, PhD
Department of Clinical Pharmacy and Pharmaceutical Care, Graduate School of Pharmaceutical Sciences, Kyushu University, 3-1-1 Maidashi, Higashi-ku, Fukuoka 812-8582, Japan
e-mail: dkobayas@med.kyushu-u.ac.jp

Supplementary Figures: 2 figures

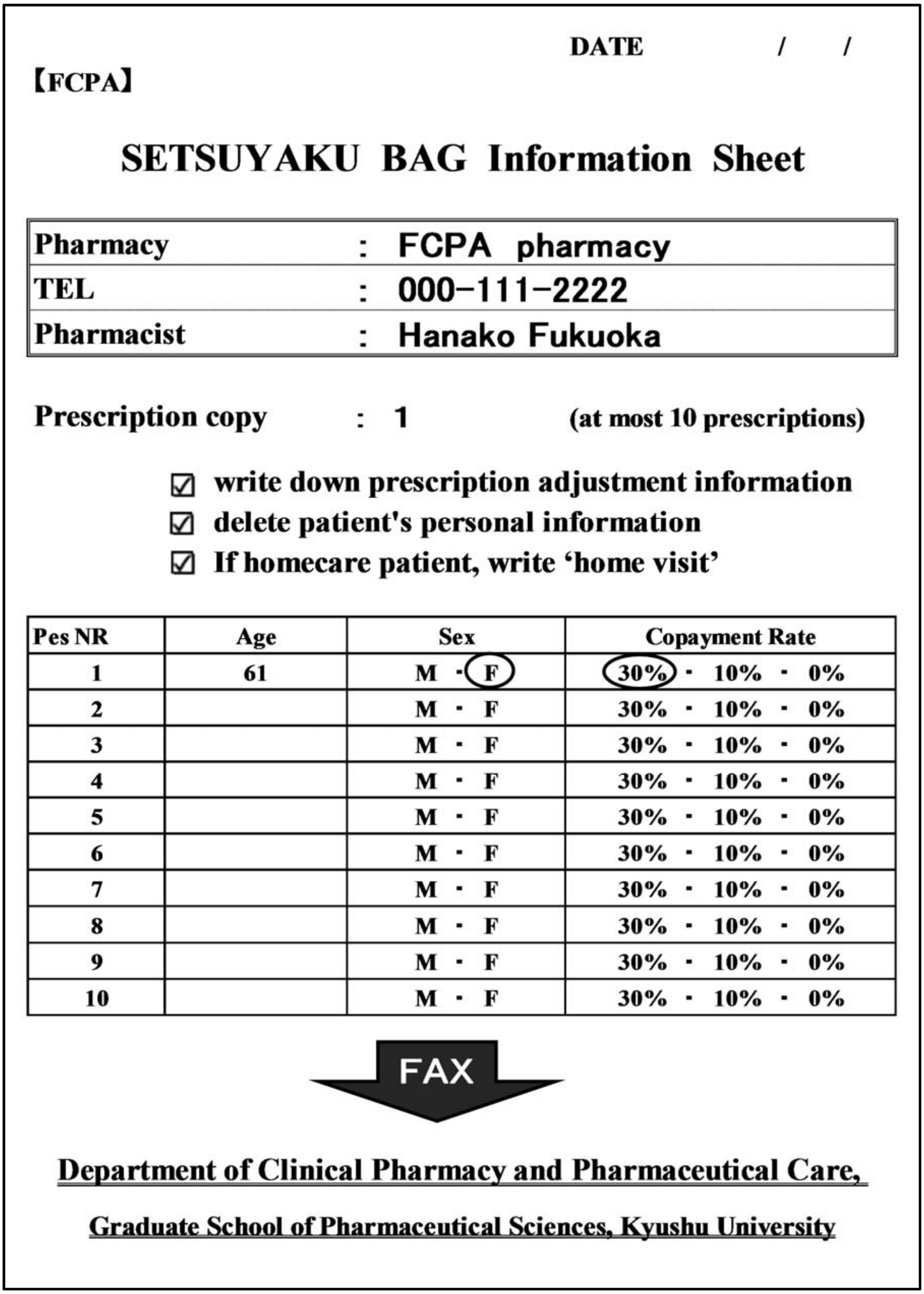

DATE / /

【FCPA】

SETSUYAKU BAG Information Sheet

Pharmacy	:	FCPA pharmacy
TEL	:	000-111-2222
Pharmacist	:	Hanako Fukuoka

Prescription copy : 1 (at most 10 prescriptions)

- ☑ write down prescription adjustment information
- ☑ delete patient's personal information
- ☑ If homecare patient, write 'home visit'

Pes NR	Age	Sex	Copayment Rate
1	61	M · F	30% · 10% · 0%
2		M · F	30% · 10% · 0%
3		M · F	30% · 10% · 0%
4		M · F	30% · 10% · 0%
5		M · F	30% · 10% · 0%
6		M · F	30% · 10% · 0%
7		M · F	30% · 10% · 0%
8		M · F	30% · 10% · 0%
9		M · F	30% · 10% · 0%
10		M · F	30% · 10% · 0%

FAX

Department of Clinical Pharmacy and Pharmaceutical Care,

Graduate School of Pharmaceutical Sciences, Kyushu University

Supplementary Figure 1. SETSUYAKU-BAG campaign information sheet for faxing.

Copayment Rate: 30%

Prescription

Public insurance No.							
Public insurance Acceptance No.							

Insurance No.	[redacted]
Insurance Detail No.	[redacted]

Patient's Name	[redacted]		
Birth Date	[redacted]		
Age	**61**	Sex	**F**
Medical Category	[redacted]		

Medical Institution Address & name

Tel

Doctor Name

[redacted]

Prescribing Date	/ /	Expiration date of Prescription	Submit it to the pharmacy within four days including the prescribing day

Formulation 1 Sitagliptin 50 mg 1 tablet / day
once in the morning 28 days ⇒ 24 days

Formulation 2 Metformin 250 mg 4 tablets / day
twice after breakfast and dinner 28 days ⇒ 21 days

Formulation 3 Voglibose 0.2 mg 3 tablets / day
three times just before each meal 28 days ⇒ 17 days

*** Situation**

This patient consults her family doctor every 4 weeks.
This time, she brought the following leftover drugs:

**Sitagliptin 50 mg**	**4 tablets**
**Metformin 250 mg**	**28 tablets**
**Voglibose 0.2 mg**	**33 tablets**

The pharmacist would prepare the drugs as follows: ⇒ Adjusted amounts

**Sitagliptin 50 mg**	**28 tablets**	**(⇒ 24 tablets)**
**Metformin 250 mg**	**112 tablets**	**(⇒ 84 tablets)**
**Voglibose 0.2 mg**	**84 tablets**	**(⇒ 51 tablets)**

Since there were usable leftover drugs,
the pharmacist adjusted the prescription as in Formulation 1-3.

Supplementary Figure 2. Copy of a prescription showing medication adjustment information (patient details have been obscured).
